# Pneumocystis jirovecii pneumonia increases the 3-months mortality of anti-MDA5-antibody-positive dermatomyositis patients

**DOI:** 10.3389/fimmu.2024.1504380

**Published:** 2024-11-28

**Authors:** Congcong Gao, Gaohui Wei, Chunyi Zhang, Chenqiong Wang, Chaoying Li, Ruxu Li, Zhaohui Su, Zhaohui Zheng

**Affiliations:** ^1^ Department of Rheumatology, The First Affiliated Hospital of Zhengzhou University, Zhengzhou, China; ^2^ Department of Clinical Laboratory, The First Affiliated Hospital of Zhengzhou University, Zhengzhou, China

**Keywords:** pneumocystis jirovecii pneumonia, anti-melanoma differentiation-related gene 5, dermatomyositis, risk factor, mortality

## Abstract

**Background:**

Anti-melanoma differentiation-associated gene 5 antibody-positive dermatomyositis (anti-MDA5+DM) patients are associated with considerable mortality, and opportunistic infections including Pneumocystis jirovecii pneumonia (PJP)is the main cause. This study was to identify clinical characteristics, risk factors, and prognostic factors of PJP diagnosed by bronchoalveolar lavage fluid (BALF) metagenomic next-generation sequencing (mNGS) in anti-MDA5+ DM patients.

**Methods:**

In this retrospective observational study, all patients admitted with suspected pneumonia were detected for mNGS in BALF. The demographics, comorbidities, laboratory parameters, and treatments of the patients were compared and analyzed in both groups to identify the potential risk factors for PJP and death via Logistic regression and Cox proportional hazards regression, respectively.

**Results:**

Overall, 92 patients were included in this study, 46(50.0%) were defined as PJP+ group, and the other 46 (50.0%) as PJP- group, and 31(67.4%) PJP occurred in the first 3 months. Increased neutrophil-lymphocyte ratio (NLR) and CRP were independent risk factors for PJP occurrence, while trimethoprim-sulfamethoxazole (TMP/SMZ) prophylaxis was an independent protective factor (all p<0.05). The three-months mortality rate was higher in the PJP+ group compared to PJP- group (43.5% vs 23.9%, p=0.047). Rapidly progressive interstitial lung disease (RPILD) was a main predictor of mortality in anti-MDA5+DM patients with PJP, whereas glucocorticoid use was a significant protective factor.

**Conclusions:**

PJP has high prevalence and mortality in anti-MDA5+DM, while TMP/SMZ prophylaxis significantly reduces PJP risk. Mortality in PJP+ patients is primarily concentrated within the first 3 months, associated with RPILD. Early intervention with corticosteroids and prophylactic measures are crucial in reducing mortality.

## Introduction

Pneumocystis jirovecii pneumonia (PJP) is a common opportunistic infection among immunocompromised individuals, including those with HIV infection, malignancy, and autoimmune diseases, posing significant morbidity and mortality risks (1). Dermatomyositis (DM) is a systemic autoimmune disorder characterized by muscle weakness and skin rash (2). Recent research has revealed an association between DM and increased susceptibility to infections, including PJP (3). Moreover, a longitudinal study spanning 13 years was conducted in patients with five rheumatic diseases, found that PM/DM patients have the highest risk of opportunistic infections, with an incidence rate of 61.3 per 1000 person-years, while, the incidence rate of PJP reaching 1.76 per 1000 person-years (4).

Anti-melanoma differentiation-associated gene 5-positive dermatomyositis (anti-MDA5+DM) is a distinct subtype of DM, typically associated with rapidly progressive interstitial lung disease (RPILD), often leading to a poor prognosis (5). Patients with anti-MDA5+DM typically undergo aggressive immunosuppressive therapy, increasing their vulnerability to opportunistic infections, especially PJP (6, 7). The incidence of infections, including opportunistic ones, in anti-MDA5+ DM patients has attracted attention, underscoring the need for a deeper understanding of infectious complications in this population.

Recently, studies employing metagenomic next-generation sequencing (mNGS) techniques have provided valuable insights into the pathogenesis and epidemiology of infections in organ transplantation, hematologic malignancies, and solid tumors (8, 9). However, their application in anti-MDA5+ DM has been limited (10). Chen et al. found that the mNGS showed a satisfying diagnostic performance with a sensitivity of 100% in detecting PJP (11). In this study, we utilized mNGS technology to identify and compare clinical characteristics, risk factors, and survival status among anti-MDA5+DM patients with PJP infection.

## Methods

### Patients

In this retrospective study, patients diagnosed with anti-MDA5+ DM who had suspected pneumonia with fever, respiratory symptoms or new radiological changes on chest CT underwent BALF mNGS at the Department of the First Affiliated Hospital of Zhengzhou University from January 2020 to December 2022 were collected. A diagnosis of anti-MDA5+DM was based on the 2017 EULAR/ACR IIM classification criteria or the 2018 EMNC DM criteria (12, 13). This study was approved by the Ethics Committee of the First Affiliated Hospital of Zhengzhou University (2020-KY-522).

### Clinical data

Baseline characteristics including demographic, clinical, comorbidities, laboratory data and treatments were acquired from the patients’ electronic medical records. Patients were followed up for at least six months. Anti-MDA5 antibody was determined in the same laboratory using ELISA kits (MBL, Japan). The presence of ILD was evaluated via chest CT. Rapidly progressive ILD (RPILD) was defined as acute progressive dyspnea and hypoxemia within 4 weeks from the onset of respiratory symptoms, accompanied by aggravation of ILD on high-resolution computed tomography (HRCT) (14).Patient treatment parameters included the daily dosage of glucocorticoids (GCs) (recorded as prednisone equivalents), immunosuppressants (IS) including cyclophosphamide (CYC), calcineurin inhibitors (CNI), tofacitinib, and intravenous immunoglobulin (IVIG). PJP prophylaxis was defined as the administration of trimethoprim-sulfamethoxazole (TMP/SMZ) for > 1 month to patients without a PJP diagnosis.

### Diagnosis of PJP

The diagnosis of PJP was based on comprehensive evaluation by clinical manifestations such as fever or acute dyspnea, characteristic radiographic findings, and etiological evidence. In this study, patients needed to have positive microbiological tests by BALF-mNGS. For these patients, we invited an experienced respiratory physician to confirm the diagnosis of PJP.

### Statistical analysis

Statistical analysis was performed with IBM SPSS software (version 26.0, Armonk, NY, USA) and GraphPad Prism software version 9.0 (GraphPad Software, San Diego, California, USA). Continuous variables with normal distributions were presented by mean ± standard deviation and those with non-normal distributions were described by median and quartiles. The independent-sample T-test and Mann-Whitney U test were used for continuous variables, while the Chi-squared test and Fisher’s exact test were used to compare categorical variables. Logistic regression models were performed to identify the independent risk factors of PJP. Covariates with a P<0.05 in the univariate analysis were included in the final model of multivariable logistic regression. Cox proportional hazards regression was used to identify independent risk factors for death. For all analyses, two-sided P-values < 0.05 were considered statistically significant.

## Results

### Characteristics

**Figure 1:**
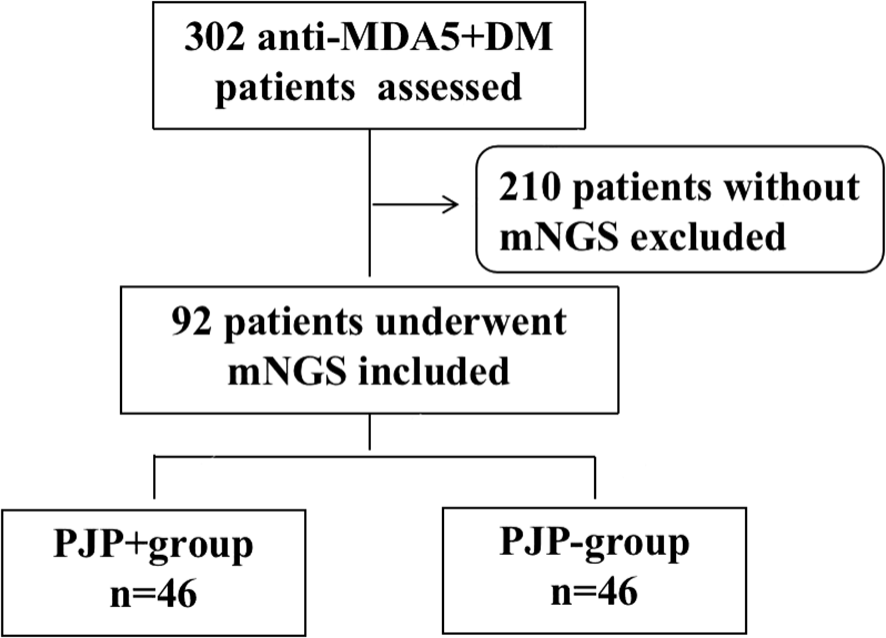
Flow chart of analyzed patients. anti-MDA5+DM:anti-melanoma differentiation-associated gene 5 antibody-positive dermatomyositis; mNGS: metagenomic next-generation sequencing.

From January 2020 to December 2022, we reviewed 302 cases with anti-MDA5+ DM from the departments of rheumatology, respiratory and ICU at the First Affiliated Hospital of Zhengzhou University. A total of 210 patients without BALF-mNGS were excluded, and 92 patients who had suspected pneumonia with fever, respiratory symptoms or new radiological changes on chest CT underwent mNGS were included in the analysis. Based on the sequencing result, patients were divided into PJP+ group (n=46) and PJP- group(n=46). All of the 92 patients were with ILD. The screening process of the patients were shown in **Figure 1**. 31.5% of these patients were newly diagnosed. The demographic features, clinical manifestations, comorbidities, laboratory data and treatments between two groups was recorded in **Table 1**. The two groups had similar ages and gender distributions, with the majority being female. Disease duration, clinical symptoms, comorbidities and treatment strategies don’t have significant difference between the two groups, except for fever, which was more prevalent in PJP+ patients (69.6% vs. 43.5%, p=0.012). Laboratory findings revealed significant differences between the groups. PJP+ patients exhibited higher neutrophil counts, lower lymphocyte counts, elevated neutrophil-to-lymphocyte ratios, and higher levels of lactate dehydrogenase (LDH), C-reactive protein (CRP), and β-D-glucan (BDG) compared to PJP- patients. Additionally, PJP+ patients had a higher rate of intensive care unit (ICU) admissions (60.9% vs. 39.1%) and were less likely to receive trimethoprim/sulfamethoxazole (TMP/SMZ) prophylaxis (8.7% vs. 30.4%). Neither of the groups received methylprednisolone pulse therapy.

**Table 1:** Characteristics of PJP+ vs. PJP- patients with anti-MDA5+DM.

Variables	PJP+group (n=46)	PJP-group (n=46)	P value
Age, years, mean(SD)	56.1 ± 12.0	53.4 ± 12.4	0.279
Male,n%	19 (41.3)	18 (39.1)	0.832
Disease course,months, median(IQR)	2 (1-4)	3 (1-4)	0.737
Newly diagnosed,n%	12 (26.1)	17 (37.0)	0.262
Clinical symptomos, n%			
Specific rash	27 (58.7)	30 (65.2)	0.519
Fever	32 (69.6)	20 (43.5)	0.012
Dyspnea	29 (63.0)	26 (56.5)	0.524
Comorbidity, n%			
Hypertension	11 (23.9)	9 (19.6)	0.613
Diabetes mellitus	9 (19.6)	8 (17.4)	0.788
Malignancy	2 (4.3)	2 (4.3)	1
RPILD	25 (54.3)	22 (47.8)	0.532
Pneumomediastinum	6 (13.0)	5 (10.9)	0.748
Laboratory indicators			
NE,×10^9^/L, median(IQR)	4.32 (2.40-5.04)	6.13 (4.80-7.51)	<0.001
LY,×10^9^/L, median(IQR)	0.38 (0.25-0.67)	0.60 (0.35-0.86)	0.007
NLR,median(IQR)	16.38 (9.37-25.88)	6.59 (3.13-13.96)	<0.001
LDH,U/L, median(IQR)	482.5 (373.5-582.3)	346 (276-434.5)	<0.001
CK,U/L, median(IQR)	45 (27.8-105)	66 (42-145.5)	0.076
ALT,U/L, median(IQR)	36.5 (27.5-84.5)	38 (24.5-64.5)	0.609
AST,U/L, median(IQR)	50.5 (41-100.5)	56 (31-98.5)	0.637
ALB,g/L,mean(SD)	30.6 ± 4.4	31.6 ± 4.6	0.289
BDG positive,n%	20 (43.5)	9 (19.6)	0.014
FET,ng/mL, median(IQR)	1127.6 (737.1-1825.5)	1025 (726.2-1781)	0.395
ESR,mm/h, median(IQR)	45.5 (29-73)	27 (21.5-66.5)	0.078
CRP,mg/L, median(IQR)	28.0 (8.2-45.2)	9.5 (1.6-21)	0.001
CD4+T-cell count, ×10^6^/L, median(IQR)	115.7 (63.6-221.5)	179 (125.8-295.5)	0.001
ICU admission, n%	28 (60.9)	18 (39.1)	0.037
IVIG,n%	35 (76.1)	34 (73.9)	0.81
TMP/SMZ prophylaxis, n%	4 (8.7)	14 (30.4)	0.009
Three-months mortality, n%	20 (43.5)	11 (23.9)	0.047
Six-months mortality, n%	24 (52.2)	19 (41.3)	0.296
GCs use,n%	34 (73.9)	29 (63.0)	0.262
GCs dosage, mg/d, median(IQR)	30 (0-56.25)	20 (0-40)	0.105
IS	21 (45.7)	16 (34.8)	0.288
CNI	15 (32.6)	15 (32.6)	1
CYC	12 (26.1)	6 (13.0)	0.115
JAKi	5 (10.9)	3 (6.5)	0.459
IS≥2	11 (23.9)	8 (17.4)	0.44

PJP, Pneumocystis jirovecii pneumonia; anti-MDA5+DM, anti-melanoma differentiation- associated gene 5 antibody-positive dermatomyositis; SD, standard deviation; IQR, interquartile range; RPILD, rapidly progressive interstitial lung disease; NE, neutrophil count; LY, lymphocyte count; NLR, neutrophil/lymphocyte ratio; LDH, lactate dehydrogenase; CK, creatine kinase; ALT, Alanine aminotransferase; AST, Aspartate aminotransferase; ALB, albumin; BDG, serum β-D-glucan; FET, ferritin; ESR, erythrocyte sedimentation rate; CRP, C-reactive protein; TMP/SMZ, trimethoprim-sulfamethoxazole; IVIG, intravenous immunoglobulin; GCs, glucocorticoids; IS, immunosuppressants; CNI, calcineurin inhibitors; CYC, cyclophosphamide; JAKi, Janus kinase inhibitors.

### Risk factors for PJP in anti-MDA5+ DM patients

**Table 2** presents the risk factors for PJP occurrence in anti-MDA5+DM patients analyzed through both univariable and multivariable models. In the univariable analysis, age, male gender, disease course, RPILD, CD4+ T-cell count, LDH, and IS showed no significant association with PJP occurrence (p>0.05). Significant risk factors included fever (OR=2.971, 95% CI: 1.261-7.00, p=0.013), NLR (OR= 1.079, 95% CI: 1.026-1.135, p=0.003), BDG positivity (OR=3.162, 95% CI: 1.244-8.039, p =0.016), CRP (OR=1.030, 95% CI: 1.010-1.051, p=0.004), and ICU admissions (OR=2.420, 95% CI: 1.047-5.590, p=0.039). TMP/SMZ prophylaxis was associated with a reduced risk of PJP (OR=0.218, 95% CI: 0.065-0.725, p=0.013). In the multivariable analysis, NLR (OR=1.096, 95% CI: 1.030-1.165, p=0.004), CRP (OR=1.034, 95% CI: 1.006-1.063, p=0.019), and TMP/SMZ prophylaxis (OR=0.030, 95% CI: 0.003-0.266, p=0.002) remained significant, while fever, BDG positivity, and ICU admissions demonstrated borderline significance.

**Table 2:** Risk factors for PJP occurrence in anti-MDA5+ DM patients.

Variables	Univariable		Multivariable	
	OR (95% CI)	P value	OR (95% CI)	P value
Age	1.019 (0.985, 1.055)	0.278	*	*
Male	1.095 (0.476, 2.520)	0.832	*	*
Disease course	0.934 (0.826, 1.056)	0.276	*	*
Fever	2.971 (1.261, 7.00)	0.013	2.740 (0.898, 8.360)	0.077
RPILD	1.299 (0.572, 2.947)	0.532	*	*
NLR	1.079 (1.026, 1.135)	0.003	1.096 (1.030, 1.165)	0.004
CD4+ T-cell count < 200 ×10^6^/L	2.095 (0.892, 4.921)	0.090	*	*
LDH	1.003 (1.001, 1.006)	0.011	1.002 (0.999, 1.005)	0.211
BDG positive	3.162 (1.244, 8.039)	0.016	2.151 (0.611, 7.575)	0.233
CRP	1.030 (1.010, 1.051)	0.004	1.034 (1.006, 1.063)	0.019
GCs use	1.015 (0.997, 1.033)	0.098	*	*
IS	1.575 (0.680,3.646)	0.289	*	*
TMP/SMZ prophylaxis	0.218 (0.065, 0.725)	0.013	0.030 (0.003, 0.266)	0.002
ICU admission	2.420 (1.047, 5.590)	0.039	0.445 (0.123, 1.603)	0.216

*Not included in the multivariable model.

PJP, Pneumocystis jirovecii pneumonia; anti-MDA5+DM, anti-melanoma differentiation- associated gene 5 antibody-positive dermatomyositis; CI, confidence interval; OR, odds ratio; RPILD, rapidly progressive interstitial lung disease; NLR, neutrophil/lymphocyte ratio; LDH, lactate dehydrogenase; BDG, serum β-D-glucan; CRP, C-reactive protein; GCs, glucocorticoids; IS, immunosuppressants; TMP/SMZ, trimethoprim-sulfamethoxazole.

### PJP mortality in anti-MDA5+ DM patients

**Figure 2:**
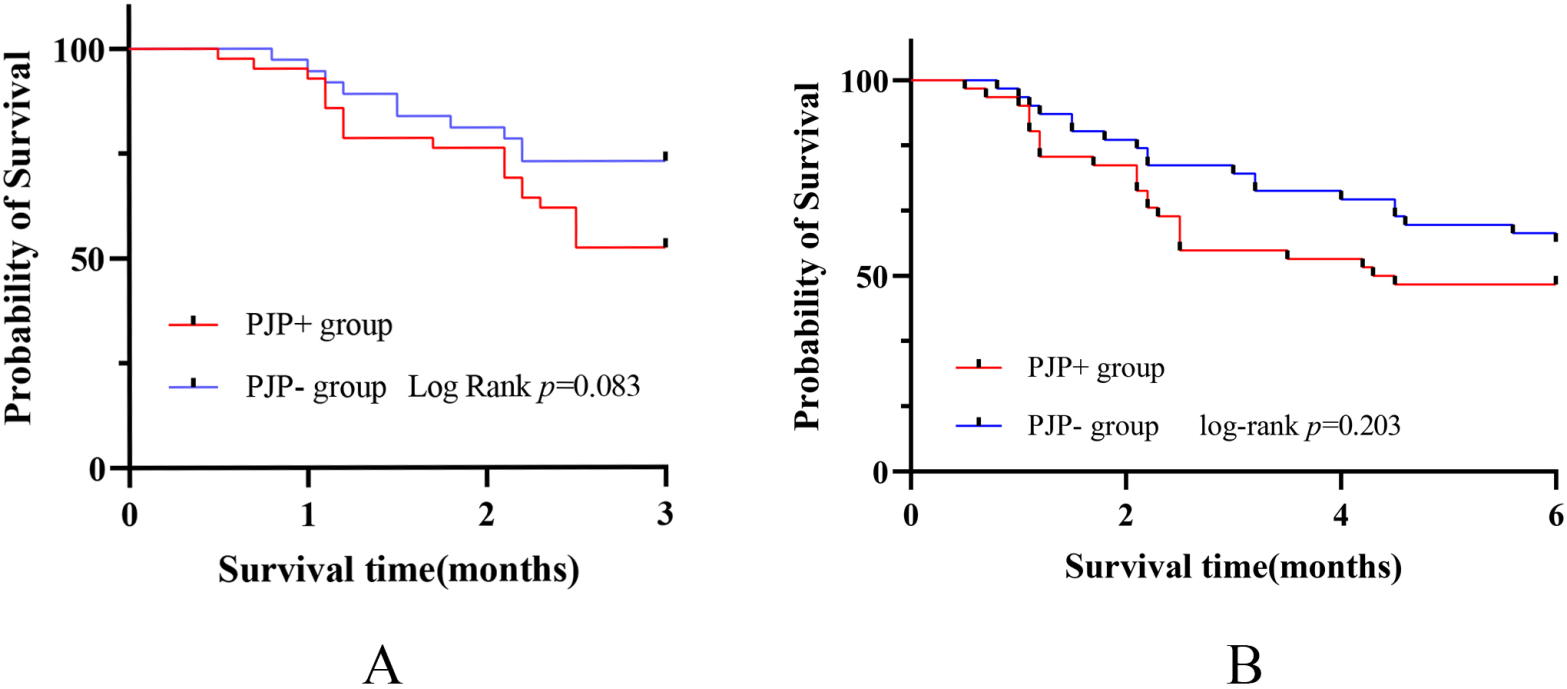
Analysis of mortality associated with PJP in anti-MDA5+ DM patients. **(A)** Kaplan-Meier curves between PJP+ and PJP- groups showed that patients with PJP had a higher mortality rate in the first 3 months. **(B)**Kaplan-Meier curves showed no statistically significant difference in mortality between the two groups within 6 months.

The 3 or 6 month mortality was calculated from the time of onset of symptoms. Notably, we found that in the first 3 months, the mortality of PJP+ group was significant higher compared to the PJP- group (43.5% vs 23.9%, p=0.047). Kaplan-Meier curves between PJP+ and PJP- groups showed that patients with PJP had a higher mortality rate (**Figure 2A**), the difference approaching statistical significance. We then used a Cox regression model to identify risk factors for mortality in patients with anti-MDA5+DM and PJP within 3 months (**Table 3**); we identified RPILD (HR: 27.602, 95%CI: 3.671-207.526), LDH (HR: 1.003, 95%CI: 1.001-1.006), and ICU (HR: 18.382, 95%CI: 2.454-137.714) as risk factors, GC use (HR: 0.141, 95%CI: 0.056-0.351) and IS (HR: 0.290, 95%CI: 0.105-0.799) as protective factors by univariate analysis. In the multivariate analysis, RPILD (HR: 14.628, 95%CI: 1.723-124.204) were ultimately confirmed as an independent risk factor, and GC use (HR:0.164, 95%CI: 0.038-0.703) were an independent protective factor.

**Table 3:** Cox regression analysis of risk factors for three-months mortality in anti-MDA5+ DM patients with PJP.

Variables	Univariable		Multivariable	
	HR (95% CI)	P value	HR (95% CI)	P value
Age	1.044 (0.994, 1.095)	0.083	*	*
Male	1.575 (0.653, 3.796)	0.312	*	*
Fever	2.073 (0.691, 6.218)	0.193	*	*
RPILD	27.602 (3.671, 207.526)	0.001	14.628 (1.723, 124.204)	0.014
NLR	1.009 (0.984, 1.033)	0.494	*	*
CD4+ T-cell count < 200 ×10^6^/L	0.576 (0.235, 1.412)	0.228	*	*
LDH	1.003 (1.001, 1.006)	0.001	1.000 (0.998-1.003)	0.735
BDG positive	1.022 (0.423, 2.468)	0.961	*	*
CRP	0.992 (0.976, 1.007)	0.295	*	*
GCs use	0.141 (0.056, 0.351)	<0.001	0.164 (0.038, 0.703)	0.015
IS	0.290 (0.105,0.799)	0.017	1.047 (0.291, 3.772)	0.944
TMP/SMZ prophylaxis	0.483 (0.065, 3.607)	0.478	*	*
ICU admission	18.382 (2.454, 137.714)	0.005	7.703 (0.949, 62.531)	0.056

*Not included in the multivariable model.

PJP, Pneumocystis jirovecii pneumonia; anti-MDA5+DM, anti-melanoma differentiation- associated gene 5 antibody-positive dermatomyositis; CI, confidence interval; HR, Hazard ratio; RPILD, rapidly progressive interstitial lung disease; NLR, neutrophil/lymphocyte ratio; LDH, lactate dehydrogenase; BDG, serum β-D-glucan; CRP, C-reactive protein; GCs, glucocorticoids; IS, immunosuppressants; TMP/SMZ, trimethoprim-sulfamethoxazole.

However, when the follow-up period was extended to 6 months, there was no statistically significant difference in mortality between the two groups(52.2% vs 41.3%, P=0.296)(**Figure 2B**). Univariate analysis showed GC use (HR:0.968, 95%CI: 0.949-0.988)) and IS (HR:0.282, 95%CI: 0.101-0.786)) were main protective factors. In the multivariate analysis, GC use (HR: 0.161, 95%CI: 0.042-0.611) was still an independent protective factor (**Table 4**).

**Table 4:** Cox regression analysis of risk factors for six-months mortality in anti-MDA5+ DM patients with PJP.

Variables	Univariable		Multivariable	
	HR (95% CI)	P value	HR (95% CI)	P value
Age	1.033 (0.968, 1.102)	0.327	*	*
Male	1.391 (0.600, 3.222)	0.442	*	*
Disease course	0.023 (0.000, 1.773)	0.089	*	*
Fever	1.750 (0.586, 5.227)	0.316	*	*
RPILD	2.406 (0.695, 8.327)	0.166	*	*
NLR	0.982 (0.956, 1.008)	0.163	*	*
CD4+ T-cell count < 200 ×10^6^/L	0.541 (0.217, 1.350)	0.188	*	*
LDH	1.002 (1.000, 1.005)	0.079	*	*
BDG positive	1.063 (0.463, 2.441)	0.886	*	*
CRP	0.985 (0.970, 1.001)	0.072	*	*
GCs use	0.968 (0.949, 0.988)	0.002	0.161 (0.042, 0.611)	0.007
IS	0.282 (0.101,0.786)	0.016	0.568 (0.164, 1.971)	0.373
TMP/SMZ prophylaxis	0.674 (0.155, 2.932)	0.599	*	*
ICU admission	3.287 (0.753, 14.359)	0.114	*	*

*Not included in the multivariable model.

PJP, Pneumocystis jirovecii pneumonia; anti-MDA5+DM, anti-melanoma differentiation- associated gene 5 antibody-positive dermatomyositis; CI, confidence interval; HR, Hazard ratio; RPILD, rapidly progressive interstitial lung disease; NLR, neutrophil/lymphocyte ratio; LDH, lactate dehydrogenase; BDG, serum β-D-glucan; CRP, C-reactive protein; GCs, glucocorticoids; IS, immunosuppressants; TMP/SMZ, trimethoprim-sulfamethoxazole.

## Discussion

Although guidelines have been published for the use of PJP prophylaxis in patients with HIV and cancer and transplant patients, there is no consensus concerning whether patients with rheumatic diseases should receive PJP prophylaxis. 2022 EULAR recommendations provide some general guidance on PJP prophylaxis in patients with autoimmune inflammatory rheumatic diseases. Daily doses >15-30mg of prednisolone or equivalent for more than 2-4 weeks, concomitant immunosuppressants, older age, underlying lung disease and persistent lymphopenia were recognized as risk factors of PJP infection, which was quite common in MDA5+DM patients (15). In this study, we offer valuable insights into the characteristics, risk factors, and mortality rates associated with PJP in patients with anti-MDA5+ DM.

PJP+ group has more fever, lower lymphocyte counts, and higher levels of CRP, and further multivariable logistic analysis suggested elevated NLR and CRP levels were independent risk factors for PJP infection, but TMP/SMZ prophylaxis was an independent protective role. However, in our study, only a small proportion of patients received TMP/SMZ prophylaxis in anti-MDA5+DM patients. The limited use of TMP/SMZ prophylaxis was manly attributed to nearly one-third of these patients being newly diagnosed. While, definitive guidelines are lacking to aid clinicians in appropriately administering prophylactic treatment. There is an urgent need for evidence-based guidelines to help healthcare providers identify specific risk factors for PJP development and initiate timely PJP prophylaxis, thus minimizing the occurrence of PJP (16).

We found that the mortality rate in the PJP+ group was significantly higher in the first 3 months compared to the PJP- group among anti-MDA5+DM patients. However, the overall mortality rate was similar at 6 months. This suggests that the primary mortality risk for PJP+ patients is concentrated in the first 3 months, which was is generally consistent with the previous report (6, 16). Therefore, for anti-MDA5+ patients, it is crucial to actively prevent PJP infection in the early stages of the disease, especially within the first 3 months. Our multivariable analysis identified elevated NLR and CRP levels as independent risk factors for PJP infection. Conversely, effective TMP/SMZ prophylaxis was shown to significantly reduce the incidence of PJP (1). Therefore, for anti-MDA5+DM patients with markedly decreased lymphocyte counts and significantly elevated inflammatory markers, early implementation of TMP/SMZ prophylaxis is recommended. Once anti-MDA5+DM patients developed PJP infection in the first 3 months, the concomitant RPILD appears to become the primary risk factor for mortality, given its significantly elevated HR. Similarity, Yoshida et al. reported that baseline lung involvement was a possible factor leading to fatal interstitial pneumonia in PJP+ rheumatoid arthritis patients (17). It is thought that the presence of underlying lung disease might create a conducive environment for opportunistic pathogens, as studies have indicated a disproportionate impact of PJP on patients with non-rheumatic pulmonary conditions (7, 18).

However, once the disease course extends beyond 3 months, the mortality risk for PJP+ patients become comparable to that of PJP- patients, and RPILD was no longer an independent risk factor for mortality, which could be linked to the stabilization of lung conditions following intensive treatment. It is worth noting that the use of corticosteroids (mean value: 30mg) can reduce the mortality rate of anti-MDA5+ patients with PJP+ at both 3 and 6 months. The debate over the use of GCs in PJP began early in the HIV epidemic (19). In 1990, several RCTs demonstrated a benefit when GCs were used in HIV patients with moderate to severe PJP (20, 21). These studies reported significant reductions in respiratory failure and death in the early stages of the disease. In non-HIV patients, the data on GCs is less clear, although some guidelines have recommended the use of GCs in PJP infection (22, 23). Retrospective studies have contradictory outcomes (24, 25); however, our results supported the use of GCs in anti-MDA5+DM patients with PJP infection. Theoretically, GCs could reduce the inflammatory response associated with PJP cell death. Some *in-vivo* studies also showed GCs had been shown to decrease cytokine release from alveolar macrophages, thereby reducing lung inflammation (26). Also, as RPILD was identified to be an independent risk factor of mortality in the MDA5+DM patients with PJP, the potential observed benefit with GCs might be contributed by therapeutic effect on RPILD.

Data concerning the effects of immunosuppressant therapy on PJP development in patients with rheumatic diseases are limited. Some IS have been reported as risk factors for PJP in some rheumatic diseases (27, 28). However, our results suggested IS were neither a risk factor for PJP infection nor do they affect the mortality rate in anti-MDA5+ DM patients with PJP infection. Recent studies have shown that early aggressive treatment is often needed, and multiagent immunosuppression is potentially more effective than traditional treatment (GC plus a single IS) (5, 29). Therefore, we suggested that the urgency of treating the primary disease should be balanced against the risk of PJP infection. When intensified treatment for the primary disease was urgently required, IS can be used without undue concern about PJP infection. Nonetheless, further large-scale cohort studies are necessary to elucidate the impact of IS on PJP infection more comprehensively.

Our study has several limitations. Firstly, it was a single-center study conducted in one hospital with a focus on a specific population, thus single-center effects cannot be ruled out. Conducting a multicenter experiment is necessary to further validate our results. Secondly, the sample size of our cohort was relatively small, which might have restricted the detection of certain potential positive outcomes. Thirdly, we only revealed risk factors for PJP occurrence in anti-MDA5+ DM patients only at one time. This is far from enough, we are conducting prospective study to explore the occurrence of PJP, and analyze the effect of PJP on anti-MDA5+ DM patients. Lastly, the high overall mortality of these patients might be attributed to more serious condition and high proportion of ICU admission. Further prospective studies with larger cohorts are warranted to confirm our findings.

## Conclusions

In this retrospective cohort study, we found that there was a high incidence and mortality in anti-MDA5+DM patients with PJP, while TMP/SMZ prophylaxis significantly reduces PJP risk. Mortality in PJP+ patients is primarily concentrated within the first 3 months, associated with RPILD. Early intervention with corticosteroids and prophylactic measures are crucial in reducing mortality.

## Data Availability

The original contributions presented in the study are included in the article/supplementary material. Further inquiries can be directed to the corresponding author.
